# Supplementary material for: Improving ALS detection and cognitive impairment stratification with attention-enhanced deep learning models
Source: Sci Rep. 2025 Feb 27;15:7045. doi: 10.1038/s41598-025-90881-9 (PMC11868594; doi:10.1038/s41598-025-90881-9)
Supplement: Supplementary file 1 — Supplementary Information. [file 41598_2025_90881_MOESM1_ESM.pdf]

Appendix

Overview

This document contains supplementary information and materials referenced in the main manuscript. Each section provides additional details to support the findings and methodologies.

1 Additional Material

1.1 Image Quality Assessment

In this study, we evaluated the quality of the dataset using several key image quality metrics to ensure that the data is suitable for further analysis and deep learning training. The following sections describe the metrics used and the corresponding violin plots, confirming that the images meet the required quality standards.

Laplacian Variance (Sharpness) Laplacian variance is used to assess the sharpness of each image in the dataset. A high Laplacian variance value indicates sharper images, while a lower value suggests blurriness. Sharp images are crucial for identifying fine details in brain tissue samples, which are essential for accurate feature extraction in deep learning models.

The violin plot for Laplacian variance demonstrates that the majority of images have high sharpness, with only a few outliers in the lower range. This distribution confirms that the dataset predominantly consists of clear and sharp images that are suitable for analysis.

Brightness Average Brightness average measures the overall intensity of each image, ensuring that the images are neither too dark nor too bright. Proper brightness is important for distinguishing between different regions and features in the images.

The brightness average violin plot shows that the images are well-distributed around a moderate brightness level, indicating that most images have appropriate lighting conditions. This ensures that brightness will not negatively affect the quality of data processing.

Contrast (Standard Deviation of Intensity) Contrast, measured as the standard deviation of pixel intensity, reflects the difference between light and dark areas in each image. Adequate contrast is necessary to highlight important features and ensure that the images contain enough detail for deep learning models to extract relevant information.

The violin plot for contrast indicates a concentrated distribution of moderate to high contrast values, suggesting that the images exhibit sufficient variability between light and dark regions. This level of contrast is optimal for deep learning tasks.

Signal-to-Noise Ratio SNR assesses the level of useful information in the image relative to background noise. High SNR values indicate clearer images with less noise, which is important for accurate classification and model performance.

The SNR violin plot shows that most images have a high SNR, with only a few low-SNR outliers. This confirms that the images are generally free from excessive noise, making them appropriate for deep learning training.

Based on the analysis of these quality metrics (Laplacian variance, brightness average, contrast, and SNR), the violin plots confirm that the dataset's image quality is acceptable. The majority of images exhibit sharpness, appropriate brightness and contrast, and low noise levels, ensuring that the dataset is valid for further data enhancement and deep learning model training.

1.2 Confusion Matrices

The confusion matrices presented in Fig.S1 illustrate the classification performance of the models across different categories. These matrices offer insights into the accuracy of predictions for each class by displaying true positives, true negatives, false positives, and false negatives, which help evaluate the model's sensitivity and specificity for each class.

1.3 Standard Formulas for Classification Metrics

In this section, we provide the standard formulas for the classification metrics used in this study: accuracy, sensitivity, specificity, and MCC. These metrics are commonly applied in the evaluation of classification models [1].

$$MCC = \frac{TP \times TN - FP \times FN}{\sqrt{(TP + FP)(TP + FN)(TN + FP)(TN + FN)}} \tag{1}$$

Accuracy represents the proportion of images correctly identified out of the overall number of predictions made.

$$Accuracy = \frac{TP + TN}{TP + TN + FP + FN} \tag{2}$$

Specificity refers to the accuracy of a test in correctly recognizing individuals who are not afflicted with the disease.

$$Specificity = \frac{TN}{TN + FP} \tag{3}$$

Sensitivity measures a test's capacity to detect individuals affected by the disease accurately.

$$Sensitivity = \frac{TP}{TP + FN} \tag{4}$$

In these formulas, TP, TN, FP, and FN stand for True Positives, True Negatives, False Positives, and False Negatives, respectively.

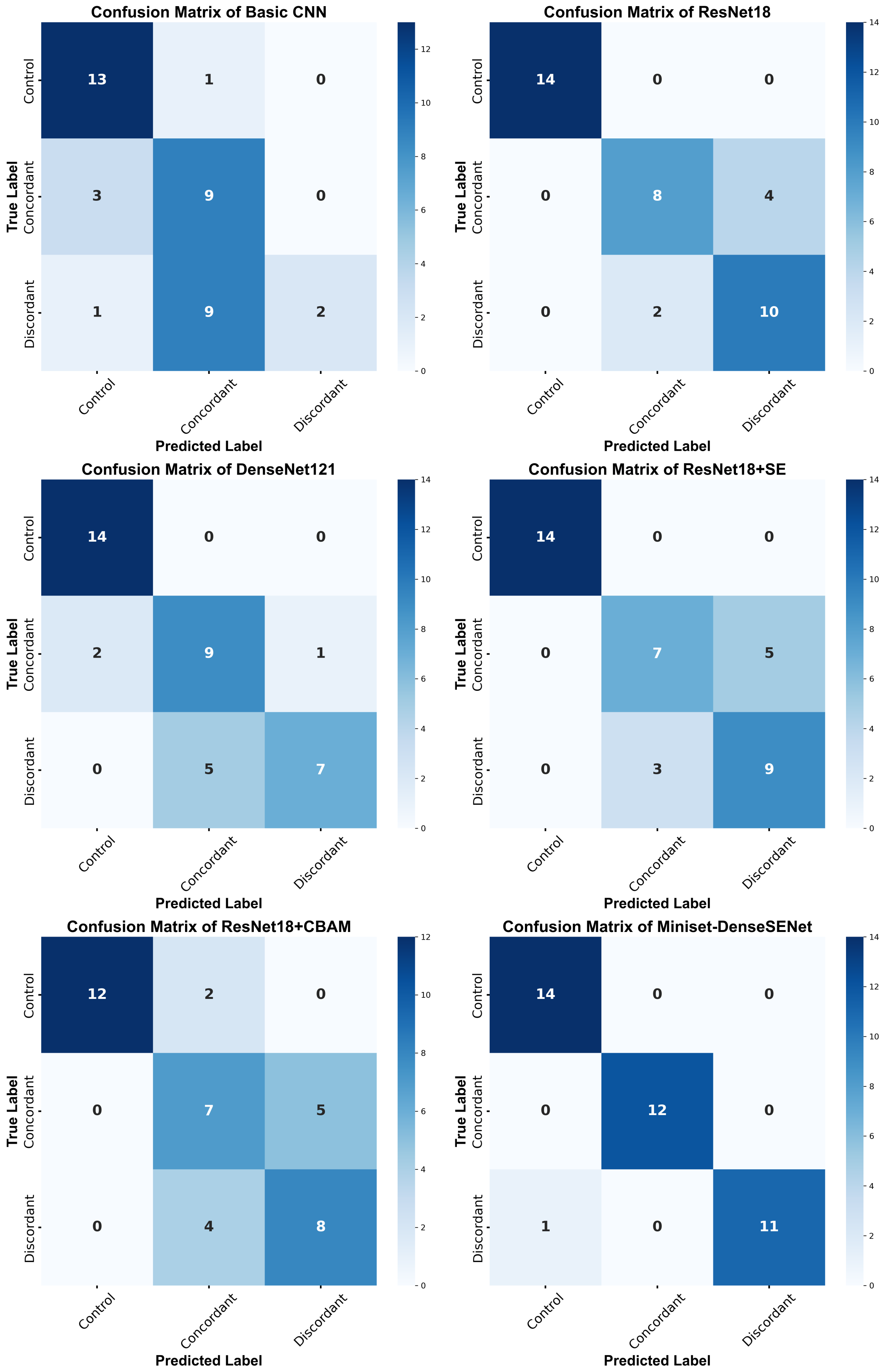

Figure S1: Confusion matrices generated by the Miniset-DenseSENet and the other CNN used for benchmarking.

References

[1] Powers, D. M. W. (2011). *Evaluation: From Precision, Recall and F-Measure to ROC, Informedness, Markedness & Correlation*. Journal of Machine Learning Technologies, 2(1), 37–63.
